# Choline intake and associations with egg and dairy consumption among pregnant women attending a high-risk antenatal clinic in South Africa: the NuEMI study

**DOI:** 10.1186/s12884-021-04314-2

**Published:** 2021-12-14

**Authors:** Liska Robb, Gina Joubert, Elizabeth Margaretha Jordaan, Jennifer Ngounda, Corinna May Walsh

**Affiliations:** 1 Department of Nutrition and Dietetics, School of Health and Rehabilitation Sciences, Faculty of Health Sciences, University of the Free State, PO Box 339, internal box G24, Bloemfontein, 9300 Free State Republic of South Africa; 2 Department of Biostatistics, School of Biomedical Sciences, Faculty of Health Sciences, University of the Free State, Bloemfontein, Republic of South Africa

**Keywords:** Choline, Pregnancy, South Africa, Birth outcomes

## Abstract

**Background:**

The importance of adequate choline intake during pregnancy has been well documented, but low intake is common. Total choline intake, main food sources of choline, as well as associations between choline intake and egg and dairy consumption were determined in a sample of pregnant women attending the high-risk antenatal clinic at a regional hospital in Bloemfontein, South Africa.

**Methods:**

A cross-sectional study design was used. Trained fieldworkers collected dietary intake data using a validated quantified food frequency questionnaire (QFFQ), after which all food items were matched to foods in the USDA Database for the Choline Content of Common Foods (Release 2) to quantify choline intake. Logistic regression with backward selection (*p* < 0.05) was used to determine whether egg and dairy consumption were independently associated with a choline intake below the adequate intake (AI) level.

**Results:**

The median daily intake of choline was 275 mg (interquartile range 185 mg – 387 mg) (*N* = 681). Most participants (84.7%) consumed less than the AI of 450 mg/day for choline. Meat and meat products, cereals, eggs and dairy contributed mostly to choline intake. Food items that contributed most to choline intake included full-cream milk, maize porridge, brown bread, deep-fried potatoes and deep-fried dough (vetkoek). A choline intake below the AI was significantly associated with lower egg and dairy intakes (*p* < 0.0001 and *p* = 0.0002 respectively).

**Conclusion:**

Most pregnant women in the current study had choline intakes below the AI. It is recommended that public health messaging targeted at pregnant women promote the consumption of foods that can significantly contribute to choline intake, such as eggs and dairy.

## Background

Choline is an important nutrient involved in human metabolism. Essential functions include structural functioning and signalling of cell membranes, methyl group metabolism, neurotransmission, and transport and metabolic processes of lipids and cholesterol [1]. Consequences of choline deficiency include the development of non-alcoholic fatty liver disease (NAFLD), liver damage possibly caused by liver cell apoptosis [2], and muscle damage [3]. Since there is insufficient data to set an estimated average requirement (EAR) or a recommended dietary allowance (RDA), dietary intake of choline is compared with the adequate intake (AI) level. The AI for choline during pregnancy is set at 450 mg, as determined by the Institute of Medicine (IOM) [4], while the European Food Safety Authority (EFSA) set an AI of 480 mg [5]. For adult females, the IOM AI is 425 mg, and for adult males, 550 mg. The AI was set at a level required to prevent liver damage [4]. The lack of EAR values for choline introduces certain limitations in the conclusions that can be drawn when populations consume choline either below or above the AI.

Choline is an essential nutrient during pregnancy, as its roles in methyl group metabolism, gene expression, membrane synthesis, tissue production, nerve transmission and brain development can have short and long-term implications in the offspring [6]. Thus, the importance of consuming adequate amounts of choline during pregnancy is becoming more apparent as more research becomes available to support this recommendation. Foods such as eggs and dairy contribute substantially to choline intake. Lewis et al. (2014) have reported that egg and milk consumption in pregnant women in Canada significantly increased the odds of consuming adequate amounts of choline [7]. Meat and liver are also excellent sources of choline but might not be as affordable and acceptable for many individuals to consume regularly. Even though meat provides a number of important nutrients, a high consumption of red and processed meats has been associated with increased mortality [8]. Meat production processes can also have negative environmental effects as a result of methane, carbon dioxide and nitrous oxide production [9].

Epidemiologic studies have shown that a choline intake below the AI is common. Vennemann et al. (2015) investigated the dietary intake of choline in European populations and found an average choline intake range of 291 to 468 mg/day in adults [1]. In the United States of America, Wallace & Fulgoni (2016) determined mean choline intake ranges for adult women (250–280 mg/day) and adult men (387–407 mg/day) [10]. These authors established that, during the period of 2009 to 2012, the estimated mean total daily dietary intake of choline in all ages ≥2 years, was 317 ± 1.8 mg. The authors concluded that the inadequate intake of choline is a public health cause of concern. In Cape Town, South Africa, Carter et al. (2018) found a median daily choline intake of 273.9 mg (using 3 × 24 h-recalls at three intervals), and 288.8 mg (using 3 x quantified food frequency questionnaires at three intervals) in heavy drinking pregnant women, also indicating an intake below the AI [11].

There is a paucity of data regarding the choline intake of populations in developing countries such as South Africa, and more research is needed to inform South African policies, guidelines and recommendations. It is also important to determine which specific food groups and foods contribute to choline intake in South Africans, as this may differ from populations in other countries.

The aim of this study was to determine choline intake, main food sources of choline, as well as associations between choline intake and egg and dairy consumption among pregnant women attending the antenatal clinic at a regional hospital in Bloemfontein, South Africa.

## Methods

### Study design, population, sample and data collection

A cross-sectional study design was implemented. All pregnant women attending the antenatal clinic at Pelonomi Hospital, Bloemfontein, South Africa, from May 2018 to April 2019 were eligible to participate in this research study. This clinic is considered a high-risk clinic to which older women (> 35 years), multiple pregnancies, women with previous poor pregnancy outcomes (neonatal death and preterm delivery), women with two or more previous caesarean sections, women with a gravida of six or more pregnancies, as well as those with obesity, hypertension or diabetes mellitus are referred from surrounding areas and towns. Information regarding the study was explained to all participants by a fieldworker. A consecutive convenience sample of 682 adult (≥ 18 years) pregnant women that participated in the Nutritional status of Expectant Mothers and their newborn Infants (NuEMI) study in their second and third trimesters who provided written informed consent, were included. In addition to other information, dietary intake data were obtained from 681 participants.

### Dietary intake tools

A quantitative food frequency questionnaire (QFFQ) was applied to obtain dietary intake information. The QFFQ was previously validated for the population in the Transition and Health during Urbanisation of South Africans (THUSA) study [12] as well as for the Women’s Health Study in the Free State, South Africa [13] and has proven reproducibility [13–15] . This QFFQ was used amongst pregnant women in the Nutrition during Pregnancy and Early Development (NuPED) study conducted in South Africa [16] with minor changes to ensure participants understood the terminology, as some vernacular differences were observed. The QFFQ comprises information about approximately 329 commonly consumed food items; however, fieldworkers in the current study could add any food item in spaces made available on the QFFQ for items not already listed. Dietary intake was determined for the previous 28 days. Nutrient intake values were divided by 28 to calculate daily intake.

In addition to food intake, participants were questioned about types and frequency of use of micronutrient supplements that were used, both from the antenatal clinic and store-bought, using a separate questionnaire.

### Techniques

Structured interviews were conducted with participants in English, Afrikaans or Sesotho which were the three most commonly spoken languages in the region in order to enhance participants’ understanding. Structured questionnaires were completed by trained fieldworkers in a designated private area within the antenatal clinic. Researchers received comprehensive training on how to obtain dietary information using a QFFQ by a South African expert in the field of dietary intake assessment from the South African Medical Research Council (SAMRC). Thereafter, fieldworkers were trained by the researchers to administer the QFFQ.

Food photographs, product packaging and a dietary intake estimation kit were used to assist participants in recalling food portion sizes and food choices. Food photographs that were used were obtained from the Dietary Assessment and Education Kit (DAEK) developed by the SAMRC, which contains photos of foods that are commonly consumed in South Africa, as well as information regarding food quantities [17]. Photos of other foods that are commonly consumed in the region were added, if not available in the DAEK. The rest of the kit comprised various household measurement tools, such as different spoons, cups, mugs, plates and bowls, all with known volumes that were available in local shops. The production of sets of beanbags with known volumes (ranging from 30 ml – 500 ml) was commissioned specifically for this study to assist participants and fieldworkers to determine portion sizes. Information related to food preparation methods and different brands of foods were collected where applicable.

Only micronutrient supplements that were provided as part of general antenatal care at the clinic (iron, folic acid and calcium) were considered in nutrient intake analyses, as information regarding privately bought supplements was not sufficiently available and was very seldom reported. Information was used to calculate daily average supplemental intake values of iron, calcium and folic acid as provided by the clinic at no cost, while no other privately obtained supplements were included in the analysis.

### Data analysis

Dietary intake data for each food code were converted to a total gram amount for the preceding 28 days and coded by the researchers (two registered dietitians). Resources used to assist researchers with calculations, included the Food Composition Tables of South Africa [18] and the SAMRC Food Quantities Manual [19]. In order to ensure consistent coding between the researchers, a coding list was developed which contained conversion factors (ml to gram), as well as portion sizes of food items not listed in the QFFQ. The two researchers that were responsible for coding met regularly to discuss and update this list.

Dietary intake data obtained from the QFFQ (daily intake of each food in grams was summarised for each participant in a Microsoft Excel file) were analysed for daily nutrient intake by the Biostatistics Unit at the SAMRC, using the South African Food Composition Database (SA-FCDB). Unlikely values (for example, very low or very high values) for nutrient intakes were flagged by assessing intake ranges of each nutrient separately, as well as checking for individual outliers, as this assisted in finding and correcting coding errors.

Choline intake was determined using data obtained from the QFFQ. The SA-FCDB could not be used, as it does not contain data for choline; thus, all food items were first matched to foods in the USDA Database for the Choline Content of Common Foods (Release 2) [20] using the FAO/INFOODS Guidelines for Food Matching [21]. Information on recipe composition was obtained from the SA-FCDB. The matching quality criteria are summarised as follows:

1. ‘High quality match (A)’: food and all its descriptors from the food consumption survey (reported food) match exactly with food and all its descriptors from the FCDB.
2. ‘Medium quality match (B)’: matching the reported food to several food items FCDB and calculating the mean food component values; or applying recipe calculations using the appropriate nutrient retention and yield factors; or matching the food item with a similar food of similar origin and calculate the mean of at least three different entries.
3. ‘Low quality match (C)’: the food is very different, but it is the closest match possible.

The choline content per 100 g of food for each food item included in the QFFQ was then determined.

Choline intake was compared to the 1998 dietary reference intake (DRI) values set by the IOM. Although the EAR should be used to evaluate population intakes of nutrients, no EAR values are yet available for choline, and thus the AI value for pregnant women (19–70 years) of 450 mg/day and the tolerable upper intake level (UL) for all adults of 3500 mg/day [4] were used in this study.

In terms of food sources and main contributors of choline, all reported food codes were assigned to a specific food group according to the designation indicated in the SA-FCDB (Table 1).

**Table 1:** Food groups indicated in the SA-FCDB

Food groups indicated in the SA-FCDB	
1. Cereal and cereal products	10. Fats and oils
2. Vegetables	11. Sugar, syrups and sweets
3. Fruit	12. Soups, sauces, seasonings and flavourings
4. Legumes and legume products	13. Beverages
5. Nuts and seeds	14. Baby foods
6. Milk and milk products	15. Therapeutic / special / diet products
7. Eggs	16. Miscellaneous
8. Meat and meat products	17. Composite dishes / recipes
9. Fish and seafood	

In order to determine the association between choline intake with egg and dairy intake, all codes related to whole egg consumption and dairy products were converted to exchanges. For eggs, food amounts of codes for boiled, fried, poached and scrambled eggs were summed, and divided by 50 g (i.e. an average egg) to determine the number of eggs consumed in the previous 28 days. For dairy, food amounts for codes for all types of liquid milk, yoghurt and *maas* (cultured milk) were summed and divided by 250 g (1 cup) to determine the number of cups of dairy consumed during the previous 28 days.

### Statistical analysis

Statistical analysis was performed by the Department of Biostatistics, Faculty of Health Sciences, University of the Free State. Data were analysed using SAS/STAT software, Version 9.4 of the SAS system for Windows, Copyright© 2013 SAS Institute Inc. SAS and all other SAS Institute Inc. product or service names are registered trademarks or trademarks of SAS Institute Inc., Cary, NC, USA [22].

Descriptive statistics, including frequencies and percentages for categorical data, and means and standard deviations (SDs) for symmetrical numerical variables, or medians and percentiles for skew numerical variables were calculated. Differences between groups were assessed by *p*-values (t-tests for symmetrical numerical variables, Mann-Whitney tests for skew numerical variables, chi-squared tests for categorical variables or Fisher’s exact tests for categorical variables with sparse data) or 95% confidence intervals (CIs) for median, mean or percentage differences. Logistic regression with backward selection (*p* < 0.05) was used to determine whether egg and dairy consumption were independently associated with a choline intake below the adequate intake (AI) level. Variables with a *p*-value of < 0.15 in bivariate analysis were considered for inclusion in the model.

Daily choline intake (milligram) for each participant as well as the median contribution (%) of each food group to choline intake was calculated by the biostatistician.

## Results

The median age of the sample (*N* = 682) was 31.8 years (interquartile range 26.8–36.5 years). Median gestational age was 32.0 weeks (interquartile range 26–36 weeks).

### Choline intake

Median daily choline intake was 275 mg, with an interquartile range of 85 mg to 387 mg (*N* = 681). Most participants consumed less than the AI of 450 mg/day for choline (84.7%), while 15.3% consumed an amount between the AI and the UL (3500 mg/day). A total of 447 reported food items were matched to the USDA Database for the Choline Content of Common Foods (Release 2). Approximately half of the reported food items were matched with a ‘high quality match’ (Table 2).

**Table 2:** Quality level of the match between the reported foods and the USDA Database for the Choline Content of Common Foods (Release 2)

QUALITY OF MATCH (***N*** = 447)	n	%
A: High quality	246	55.0
B: Medium quality	172	38.5
C: Low quality	29	6.5

### Multivitamin and mineral supplement use

Few participants (2.5%) indicated that they obtained a multivitamin and mineral (MVM) supplement (which could have contained choline) from the clinic, while 4.7% obtained such a supplement from a store.

### Food and food group sources of choline

The median contribution (%) of each food group to total choline intake is presented in Table 3. In the current study, meat, cereal, eggs and dairy were the main contributors to choline intake.

**Table 3:** Median contribution (%) of each food group to total choline intake of the sample (*N* = 681)

FOOD GROUP	Median contribution (%) and IQR
Meat and meat products	24.2 (14.9–34.6)
Cereal and cereal products	22.2 (16.3–30.7)
Eggs	11.7 (0.0–25.8)
Milk and milk products	8.3 (4.9–12.9)
Vegetables	6.2 (3.9–9.6)
Fruit	2.9 (1.6–4.6)
Composite dishes / recipes	2.5 (0.75–5.7)
Fats and oil	1.0 (0.3–2.3)
Fish and seafood	0.9 (0.0–3.3)
Soups, sauces, seasonings and flavourings	0.2 (0.0–0.8)
Sugar, syrups and sweets	0.1 (0.0–0.6)
Legumes and legume products	0.0 (0.0–1.9)
Nuts and seeds	0.0 (0.0–0.4)
Beverages	0.0 (0.0–0.0)
Baby foods	0.0 (0.0–0.0)
Therapeutic / special / diet products	0.0 (0.0–0.0)
Miscellaneous	0.0 (0.0–0.0)

Table 4 presents the median choline (mg) contribution of specific food items to choline intake. The table includes all food items for which a median choline contribution of more than zero was calculated. The quality of the match with the USDA Database for the Choline Content of Common Foods (Release 2) is also indicated. Full cream milk was the main food contributing to choline intake.

**Table 4:** Median contribution (%) of each food item to choline intake as well as quality of match (*N* = 681)

Median choline contribution of specific food items to the choline intake	Median contribution (%) and IQR	Match quality
Full cream milk	4.7 (1.5–8.3)	A
Brown bread	2.5 (0.0–5.8)	A
Maize meal, stiff	1.7 (0.0–4.3)	B
Slap chips (deep fried potatoes)	1.6 (0.2–3.8)	A
Eggs, fried	1.5 (0.0–16.9)	A
Vetkoek (deep fried dough)	1.1 (0.0–3.8)	B
Boerewors (beef sausage)	0.6 (0.0–1.8)	A
Banana	0.5 (0.1–1.0)	A
Apple	0.4 (0.1–0.7)	A
Russian (fried pork sausage)	0.5 (0.0–2.0)	B
White bread	0.3 (0.0–2.7)	A
Amasi (fermented milk drink/yoghurt)	0.3 (0.0–1.9)	C
White rice	0.2 (0.1–0.3)	A
Tea	0.1 (0.0–0.3)	A
Mayonnaise, regular	0.04 (0.0–0.6)	A
Maize meal, soft	0.03 (0.0–0.6)	B
Cold drinks	0.02 (0.0–0.1)	A
Margarine, regular	0.02 (0.0–0.3)	A

### Associations between egg and dairy consumption and choline intake

More than one third of participants (36.3%) had not consumed whole eggs during the previous 28 days (Table 5), while almost one out of five participants consumed more than five eggs per week (18.2%). Most participants consumed less than one cup of dairy per day. It should be noted that although 23.2% of participants consumed more than one cup of dairy per day, the majority consumed less than two cups, and only eight participants consumed more than two cups per day (1.2%) (Table 5). A statistically significant association was found between lower egg (*p* < 0.0001) or dairy intake (*p* = 0.0002) and inadequate choline intake. Out of the 247 participants who did not consume any eggs, only five (2.0%) consumed an adequate amount of choline. No participant who did not consume any dairy managed to consume an adequate amount of choline.

**Table 5:** Associations between egg and dairy intake with choline intake

	Sample prevalence (*N* = 681)		Choline intake < 450 mg (*N* = 577)		Choline intake ≥ 450 mg (*N* = 104)		*p*-value
	n	%	n	%	n	%	
**Weekly egg intake**							**< 0.0001**
No egg intake	247	36.3	242	98.0	5	2.0	
Zero to < 1	74	10.9	71	96.0	3	4.1	
1 to < 3	133	19.5	121	91.0	12	9.0	
3 to < 5	103	15.1	79	76.7	24	23.3	
≥ 5	124	18.2	64	51.6	60	48.4	
**Daily dairy intake**							**0.0002**
No dairy intake	25	3.7	25	100.0	0	0.0	
< 250 g	498	73.1	433	87.0	65	13.1	
≥ 250 g	158	23.2	119	75.3	39	24.7	

Logistic regression with backward selection (*p* < 0.05) was used to investigate the independent association of weekly egg intake (no egg intake, zero to < 1, 1 to < 3, 3 to < 5, ≥ 5) and daily dairy intake (< 250 g, ≥ 250 g) with choline intake. The odds ratios are indicated in Table 6**.** As egg and dairy intake increased, the odds of a choline intake below the AI decreased.

**Table 6:** Weekly egg and daily dairy intake associated with inadequate choline intake: logistic regression

Variable	Description	Odds ratio (95% CI)	***p***-value
Weekly egg intake	none vs ≥ 5	49.78 (18.96; 130.68)	**< 0.0001**
	< 1 vs ≥ 5	21.82 (6.47; 73.62)	
	1 to < 3 vs ≥ 5	10.64 (5.23; 21.64)	
	3 to < 5 vs ≥ 5	3.30 (1.82; 5.98)	
Daily dairy intake	< 250 g vs ≥ 250 g	2.80 (1.64; 4.73)	**0.0002**

## Discussion

### Choline intake

The median daily choline intake from food sources was 275 mg in this sample of pregnant women. Almost all participants consumed less choline than the AI recommends. Anecdotal evidence suggests that choline is usually not added to MVM supplements, and very few brands available in most South African shops and pharmacies contain choline. A Canadian study found that none of the prenatal supplements that were used by their participants contained choline [23]. Additionally, MVM supplement use was not common in this sample; thus, it can be concluded that participants in the current study relied on food sources for choline intake. A population level choline intake below the AI has been reported by various researchers, from both developing [11, 24, 25] and developed [1, 10, 26, 27] countries. Based on the important functions of choline during pregnancy, especially related to foetal growth and development [6], the population groups that are most likely to benefit from adequate choline intake are pregnant women and their offspring.

### Food sources of choline

Choline is found in a variety of foods; however, animal products generally have higher levels of choline than plant products. Good sources of choline include eggs, beef, chicken, fish, dairy, cruciferous vegetables, certain beans [28], as well as beef liver, chicken liver, wheat germ, dried soybeans and pork [29]. In the current study, food groups that contributed most to choline intake, included meat, cereals, eggs and dairy. This is in agreement with the established good food sources of choline, as well as with research done in European populations [1].

In the current study, full-cream milk, maize meal, brown bread, fried eggs, slap chips (deep fried potatoes) and vetkoek (deep fried dough) were among the top foods that contributed to choline intake. However, individual food items that contributed most to choline intake were influenced by the amount of the food item generally consumed, not simply the choline density. Food items that are considered to be poorer sources of choline, but were consumed in high amounts, were among the top contributors of choline in this sample of pregnant women. For example, bread, maize meal, apples and bananas are not good sources of choline, but were consumed relatively frequently and in large quantities and as such, could be regarded as important sources of choline in the diet of this population. This important aspect must be considered when promoting the intake of choline in populations from different countries, as eating habits differ significantly among cultures. In addition, many of the top choline contributing foods in this sample are not generally considered to be health-promoting foods, as processed, high-fat, high-salt foods featured prominently (fried egg, deep fried dough, beef sausage, fried pork sausage, white bread, white rice, cold drinks). Although these foods might have contributed to choline intake, regular consumption thereof can contribute to obesity and other non-communicable diseases (NCDs) as well as micronutrient deficiencies in general. Intake of healthier sources of choline, such as boiled eggs, lean meat, dairy and wholegrain products should be promoted among pregnant women.

In the current study, eggs in general, and fried eggs specifically, were some of the top foods or food groups that contributed to choline intake. However, many participants did not consume any eggs. Eggs are exceptionally high in choline; thus, the regular consumption of eggs may contribute substantially to choline intake. In the current study, a choline intake below the AI was significantly associated with lower egg consumption (*p* < 0.0001), supporting this supposition. Additionally, the odds of consuming a choline intake below the AI increased substantially as egg intake decreased. In general, egg consumption in South Africa has increased by 55.8%, from 1994 to 2009, and by 24.1%, from 1994 to 2012. Per capita egg consumption in 2012 was 7.2 kg per year [30], which translates to three large eggs per person per week. The South African Food Based Dietary Guidelines (SAFBDGs) recommend an egg intake of “approximately four eggs per week” [31]. Culture and food taboos may influence the consumption of foods such as eggs, and this phenomenon is also common during pregnancy. Chakona and Shackleton (2019) investigated the influence of food taboos and beliefs on food choices among pregnant women in the Eastern Cape, South Africa. They found that eggs were among the most commonly avoided foods during pregnancy, along with meat, fish, potatoes, fruits, beans and pumpkin. The authors suggest culturally appropriate education for this population with the goal of improving nutrient intake to optimally support requirements during pregnancy [32]. Socioeconomic status and the price of eggs might also influence egg consumption. A study by Headey et al. (2017) to investigate animal sourced foods and child stunting, showed that eggs are a relatively inexpensive source of nutrients in higher income countries. However, the authors state that in Africa eggs are nine to ten times more expensive than staple cereals [33].

Although most participants consumed dairy, it was mostly consumed in relatively small amounts (< 1 cup per day). The SAFBDGs specifically recommend a daily intake of 400–500 ml low-fat milk for all adults [34]. Although, per weight, dairy products are not as high in choline as eggs, they can contribute substantially to choline intake if consumed frequently. A lower dairy intake was significantly associated with a choline intake below the AI in the current study (*p* = 0.0002), and the odds of consuming a choline intake below the AI increased as dairy intake decreased. Additionally, full cream milk was the one specific food item that contributed most to choline intake, suggesting that dairy can contribute meaningfully to choline intake in this population. Consumption of dairy in South Africa has increased by 8.4% from 1994 to 2009, and by 14.7% from 1999 to 2012. Increases were observed especially for yoghurt and cultured milk consumption, and to a lesser degree, for cheese and fluid milk [30].

Animal derived foods are generally the top contributors to choline intake in the diets of most populations. Currently, there is widespread promotion of plant-based foods, together with the recommendation of limiting animal derived foods. These recommendations are based on the prevention of NCDs as well as contributing to sustainable and environmentally friendly food production systems [35]. However, the reduction in consumption of animal derived foods may inadvertently lead to a reduction in the intake of certain important nutrients, including choline, which is an important factor to consider, specifically in pregnant women and young children. Additionally, infants and young children who consume nutritionally inadequate diets are at risk of developing malnutrition and associated hepatic steatosis [36]. Research has shown that the development of malnutrition-associated hepatic steatosis was prevented by choline supplementation in mice dams [37]. The authors concluded that sub-optimal choline intake may increase the risk of hepatic steatosis in undernutrition. In a country such as South Africa with high prevalence of undernutrition, the implications of further limiting the intake of animal protein may be far-reaching.

South Africa is a low-resource country with a high burden of both communicable and non-communicable diseases, and strategies that could contribute to an improvement in population health, warrants attention. It is recommended that intake of choline during pregnancy should be encouraged in the South African population by means of public health messaging as well as possible changes in food fortification policies to include choline. Since egg and dairy intake were significantly associated with a choline intake above the AI, the promotion of culturally acceptable egg and dairy consumption (e.g. cultured milk) during pregnancy should be supported. The supplementation of iron, folic acid and calcium during pregnancy is currently recommended in the South African Guidelines for Maternity Care [38], and as choline is such an important metabolic nutrient, especially for the mother and foetus, there could be, in the light of these results, a case to be made for the addition of choline to this regimen. This should however be considered after robust human clinical trials have shown consistent benefits with maternal choline supplementation, and thus more trials to investigate the effects of maternal choline supplementation are recommended.

#### Limitations

Due to the fact that choline is not included in the South African Food Composition Database, foods consumed in the current study had to be matched to foods in the USDA Database for the Choline Content of Common Foods (Release 2) using the FAO/INFOODS Guidelines for Food Matching [21]. These guidelines provide a food matching approach that is universally applicable. Approximately half of the food items had a high-quality match, that support the accuracy of choline intake results.

The differences in research findings regarding population-level choline intake in different countries should be interpreted with caution as most researchers will use the USDA Database for the Choline Content of Common Foods, as it is the most comprehensive. However, different releases thereof might influence the calculated choline intake results among studies. Another aspect to consider is that not all researchers use the FAO/INFOODS Guidelines for Food Matching when matching their database to the USDA database, which could cause discrepancies. Data were collected from one high-risk antenatal clinic only and cannot be extrapolated to the whole of South Africa.

Choline from all foods that were reported by the women in the study were considered to determine choline intake in the sample, but only whole eggs and dairy were independently analysed as predictors of choline intake. Meat and meat products are also considered as good sources of choline; however, due to the fact that eggs are some of the richest food sources of choline, and dairy is a major contributor to choline intake, as a result of frequent consumption, associations between egg and dairy consumption and choline intake were further studied in this population. Egg and dairy products are also generally more affordable than meat products, and may play an important role in increasing choline intake in low-resource settings.

Although the QFFQ has been used in a number of studies undertaken in South Africa, we acknowledge that it (and the items in the dietary intake estimation kit) was not specifically validated to determine choline intake in this setting. However, the QFFQ lists 329 foods which includes most commonly consumed foods. Participants could also add foods that were not listed.

## Conclusions and recommendations

Pregnancy forms part of the ‘first thousand days’, a critical period for optimal development of the foetus and infant, and adequate nutrient intake is of paramount importance. A sub-optimal choline intake during this period might have lasting negative effects on the offspring. Consumption of foods that can significantly contribute to choline intake, such as eggs and dairy, should be encouraged through public health messaging to promote optimal choline intake during pregnancy.

Since an EAR for choline is not available, it is challenging to determine the prevalence of population-level deficiencies. According to the IOM [39], if the mean intake of a nutrient is below the AI, as in the current study, the adequacy of the group’s intake cannot be reported with confidence. The EAR may be used to determine prevalence of choline inadequacy of populations; however, using the AI as a cut-point may overestimate the true prevalence of inadequacy. Consequently, even though most participants in the current study did not meet or exceed the AI for choline, and the median intake was below the AI, the conclusion of a high prevalence of inadequate intake in this population cannot be made explicitly. The absence of an EAR value leads to challenges when attempting to estimate choline deficiency in a population, thus the development of an EAR value is recommended in order to more accurately determine the prevalence of choline deficiency in general and more specifically, in pregnant women.

## Abbreviations

AI: Adequate intake
CI: Confidence interval
DAEK: Dietary Assessment and Education Kit
DRI: Dietary reference intake
EAR: Estimated average requirement
EFSA: European Food Safety Authority
FAO: Food and Agriculture Organisation
IOM: Institute of Medicine
MVM: multivitamin and mineral
NAFLD: Non-alcoholic fatty liver disease
NCD: Non-communicable diseases
NuPed: Nutrition during Pregnancy and Early Development
QFFQ: quantified food frequency questionnaire
RDA: Recommended dietary allowance
SAFBDGs: South African Food Based Dietary Guidelines
SA-FCDB: South African Food Composition Database
SAMRC: South African Medical Research Council
SD: Standard deviation; UL: tolerable upper intake level
USDA: United Sated Department of Agriculture
